# Association of BLM and BRCA1 during Telomere Maintenance in ALT Cells

**DOI:** 10.1371/journal.pone.0103819

**Published:** 2014-08-01

**Authors:** Samir Acharya, Zeenia Kaul, April Sandy Gocha, Alaina R. Martinez, Julia Harris, Jeffrey D. Parvin, Joanna Groden

**Affiliations:** Department of Molecular Virology, Immunology and Medical Genetics, College of Medicine, The Ohio State University, Columbus, Ohio, United States of America; The University of Hong Kong, Hong Kong

## Abstract

Fifteen percent of tumors utilize recombination-based alternative lengthening of telomeres (ALT) to maintain telomeres. The mechanisms underlying ALT are unclear but involve several proteins involved in homologous recombination including the BLM helicase, mutated in Bloom's syndrome, and the BRCA1 tumor suppressor. Cells deficient in either BLM or BRCA1 have phenotypes consistent with telomere dysfunction. Although BLM associates with numerous DNA damage repair proteins including BRCA1 during DNA repair, the functional consequences of BLM-BRCA1 association in telomere maintenance are not completely understood. Our earlier work showed the involvement of BRCA1 in different mechanisms of ALT, and telomere shortening upon loss of BLM in ALT cells. In order to delineate their roles in telomere maintenance, we studied their association in telomere metabolism in cells using ALT. This work shows that BLM and BRCA1 co-localize with RAD50 at telomeres during S- and G2-phases of the cell cycle in immortalized human cells using ALT but not in cells using telomerase to maintain telomeres. Co-immunoprecipitation of BRCA1 and BLM is enhanced in ALT cells at G2. Furthermore, BRCA1 and BLM interact with RAD50 predominantly in S- and G2-phases, respectively. Biochemical assays demonstrate that full-length BRCA1 increases the unwinding rate of BLM three-fold in assays using a DNA substrate that models a forked structure composed of telomeric repeats. Our results suggest that BRCA1 participates in ALT through its interactions with RAD50 and BLM.

## Introduction

Telomeres are DNA-protein complexes comprised of repetitive non-coding DNA sequences at the ends of eukaryotic chromosomes and the proteins that bind these sequences. In mammals, telomeres consist primarily of TTAGGG sequences [1]–[5]. Telomeres prevent chromosome erosion and loss of coding sequences due to the end-replication problem. Loss of telomeric DNA is linked with cellular senescence and aging, and likely resembles double-strand breaks that activate DNA damage response pathways [6]–[9]. While cell growth continuously reduces telomere length, cancer cells become immortalized by activating mechanisms of telomere maintenance. The most common mechanism is expression of the enzyme telomerase, which catalyzes the addition of repeats to maintain telomere length. Approximately 15% of human tumors maintain telomeres independently of telomerase and use a recombination-based mechanism known as alternative lengthening of telomeres (ALT) to maintain telomere lengths [10]–[17]. ALT cells are typified by the presence of ALT-associated PML bodies (APBs) that include telomeric DNA and telomeric proteins [15], [18]. Although the functions of APBs are unclear, they are considered primary sites of telomere metabolism. Aberrant telomere metabolism results in telomere dysfunction, yield chromosomal abnormalities, such as chromosome end-to-end fusions, telomeric translocations, tri- and quadri-radial chromosomes, and limit growth potential [8], [19]–[22].

The mechanisms of ALT remain unclear. However, several DNA damage response proteins are implicated in ALT due to their association with telomeres or APBs, including the recQ-like helicases BLM (defective in Bloom's syndrome) and WRN (defective in Werner's syndrome), and the tumor suppressor BRCA1 [23]–[30]. BLM inhibits recombination by facilitating the resolution of recombination and replication intermediates. Through its structure-specific unwinding activity, BLM helps to resolve DNA damage-induced replication blocks that if left unresolved will result in aberrant recombination and chromosomal breakage. BLM associates with numerous proteins involved in DNA repair including BRCA1, DNA topoisomerases, DNA mismatch repair proteins and Fanconi anemia proteins, and is a component of the BRCA1-associated genome surveillance complex (BASC) [31]–[33]. BLM also associates with several telomere-specific proteins, such as POT1, TRF1 and TRF2 [34]–[37]. Biochemically, POT1 stimulates BLM unwinding of telomeric DNA end structures including D-loops and G-quadruplexes during DNA replication and/or recombination. TRF1 and TRF2 also modulate BLM function using telomeric substrates. The role of BLM in telomere metabolism is emphasized by telomere dysfunction in cells from those with Bloom's syndrome or cells lacking BLM, including increased telomeric associations and increased frequency of anaphase bridges involving telomeres [25], [28], [38]–[40]. While BLM plays a major role in regulating genomic sister chromatid exchange, studies investigating telomeric sister chromatid exchange (T-SCE) in cells lacking BLM have yielded inconsistent results but do not support a major role for BLM in regulating T-SCEs in ALT cells [38]–[40].

The tumor suppressor BRCA1 performs a key role in the cellular DNA-damage response and recombination repair by promoting both homologous recombination and non-homologous end-joining [41]–[48]. Its recruitment to DNA double strand breaks (DSB) is facilitated by the damage sensor MRN (MRE11-RAD50-NBS1 complex) and is critical for further assembly and employment of other recombination proteins to the site. BRCA1 consists of N-terminal RING domain and C-terminal tandem BRCT domains. The RING domain mediates its interaction with BARD1. Through the BRCT domains, it forms several distinct complexes including BRCA1-Abraxas, BRCA1-BACH1 and BRCA1-CtIP that perform key roles during initiation of recombination [48]. In addition, it functions in other DNA repair pathways such as non-homologous end joining (NHEJ) and nucleotide excision repair pathways, and is also part of BASC in genome surveillance [49], [50]. Several studies implicate BRCA1 in telomere maintenance [51]–[53]. BRCA1 deficiency results in telomere dysfunction, as evidenced by elevated chromosome fusions and translocations involving telomeres and telomere shortening [52], [54]. *Brca1* ^−/−^ murine T-cells display a high incidence of telomere instability [54], while expression of dominant-negative BRCA1 in human cells results in telomere dysfunction [55]. BRCA1 interacts with the telomere binding proteins TRF1 and TRF2 and is localized to telomeres. Its over-expression inhibits telomerase transcription and promotes shortening of telomeres in telomerase-positive breast and prostate cancer cell lines [56]. Interestingly, BRCA1 knockdown increases telomere length in some telomerase-positive cells [53]. An essential role of BRCA1 in telomere length maintenance is further emphasized by recent reports on telomere length measurements in those with familial breast cancer (those carrying mutations in *BRCA1*) indicating a correlation between telomere shortening in these affecteds and age of breast cancer onset [57].

The biochemical mechanisms by which BRCA1 mediates telomere maintenance are undefined. Although BRCA1 interacts with the recQ-like helicases WRN and BLM to facilitate intra-strand cross-link repair and DSB repair [58], BRCA1 interaction with BLM in the context of telomere metabolism is unclear. This study explores the role of BLM and BRCA1 in telomere metabolism in ALT cells.

## Results, Discussion and Conclusions

### BLM and BRCA1 co-localize at ALT telomeres

**Figure 1:**
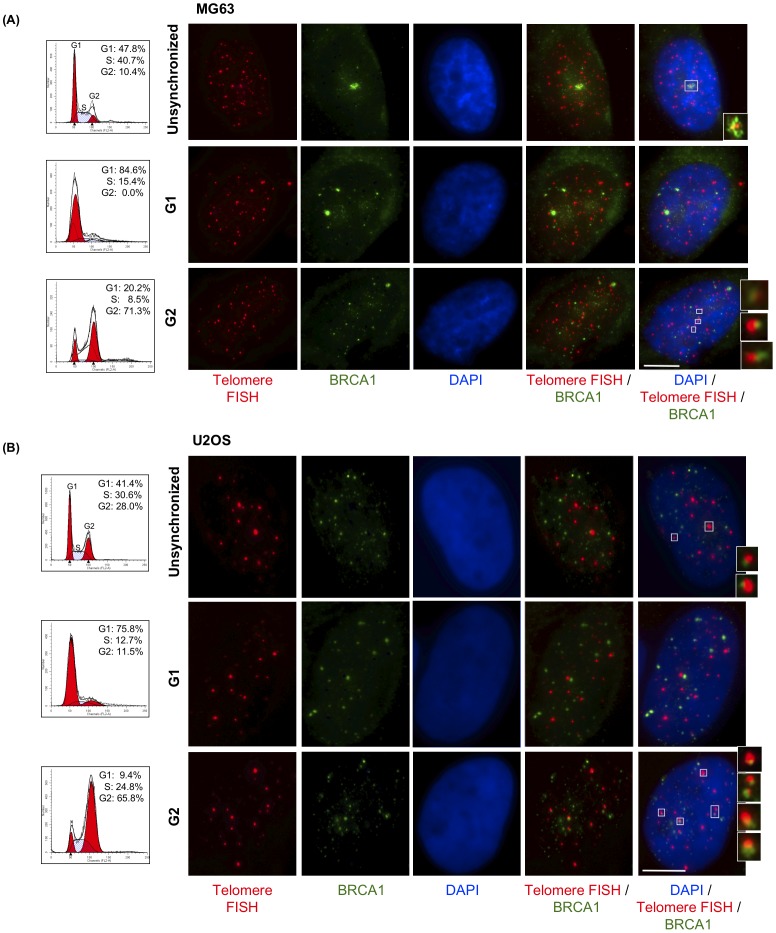
BRCA1 localizes to ALT cell telomeres primarily in G2 of the cell cycle. (**A**) MG63 and (**B**) U2OS cells were synchronized by double thymidine block. Asynchronous and synchronized cells were collected and analyzed by flow cytometry to determine cell cycle distribution. Cells were fixed at corresponding phases of cell cycle and stained with antibodies to BRCA1 (green); telomeres were labeled by FISH with a PNA probe (red); nuclei were stained with DAPI (blue). Images shown are a single maximum intensity projection (MIP) image. Enlargements of co-localizations are shown at the right. Scale bar: 10 µm.

**Figure 2:**
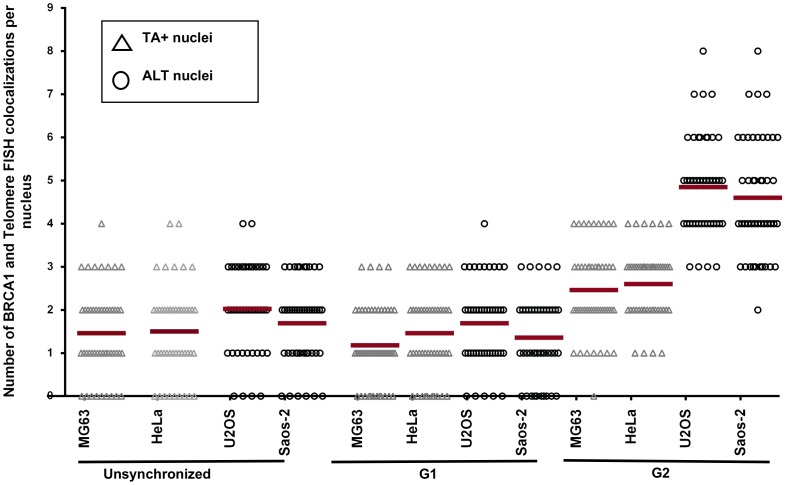
Telomeric localization of BRCA1 is increased in ALT cells. BRCA1 and telomere co-localizations in Figure 1 were quantitated per nucleus in unsynchronized, G1- and G2-synchronized MG63 (telomerase positive, TA+), HeLa (TA+), U2OS (ALT) and Saos-2 (ALT) cells. Each triangle (TA+) or open circle (ALT cells) represents an individual cell. Red bars indicate mean values.

Our recent study demonstrated that telomerase-negative tumor cell lines utilize different ALT mechanisms to maintain telomeres [59]. Some of these mechanisms depend on BRCA1 and depletion of BRCA1 in these cells results in a modest decrease in telomeric sister-chromatid exchange [59]. Depletion of BLM results in a dramatic reductions in APB formation and telomere length in all cell lines utilizing ALT, confirming the role of BLM in telomere maintenance in ALT and/or induction of ALT [59], [60]. Both BLM and BRCA1 are essential components of DNA damage-induced replication/recombination foci and are independently associated with telomere maintenance. To test for a cooperative role of BRCA1 and BLM in telomere maintenance, *in situ* localization studies were performed using telomerase-positive (TA+) cells (HeLa and MG63) and telomerase-negative/ALT cells (Saos-2 and U2OS). Cells were synchronized by double thymidine block and release to assess cell cycle-dependent localization of BRCA1 and a telomeric Cy3-(CCCTAA)_3_ PNA probe using immunofluorescence and telomere FISH. Asynchronous cells were used as controls. BRCA1 localizes to telomeres primarily in the G2 phase of the cell cycle in ALT cells (Figure 1A, B). Quantitation shows a 2.5-fold increase in telomeric localization of BRCA1 in ALT cells (∼5 foci per nucleus) compared to telomerase-positive cells (∼2 foci per nucleus) (Figure 2). Knockdown of *BRCA1* by *siRNAs* eliminates co-localization, demonstrating staining specificity (Figure S1). These results are consistent with a role for BRCA1 in telomere metabolism.

**Figure 3:**
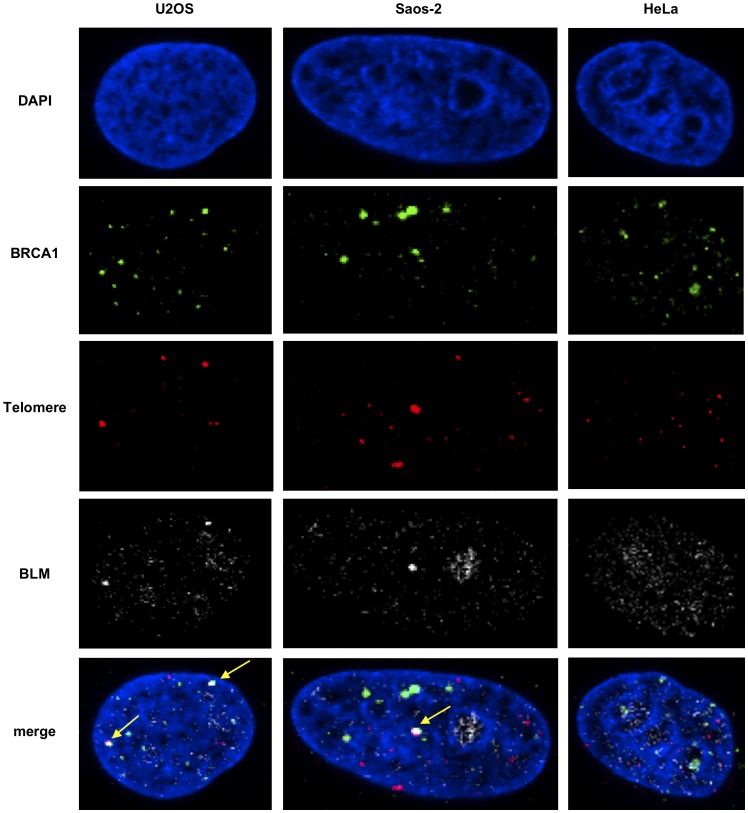
BRCA1 and BLM co-localize at telomeres in ALT cells. (**A**) Cells were synchronized to G2 (7h post release after double thymidine block) and stained with antibodies to BRCA1 and BLM and telomeres labeled by FISH with a PNA probe (upper panel) as in Figure 1. Yellow arrows indicate foci with BLM-BRCA1-telomere co-localization.

**Figure 4:**
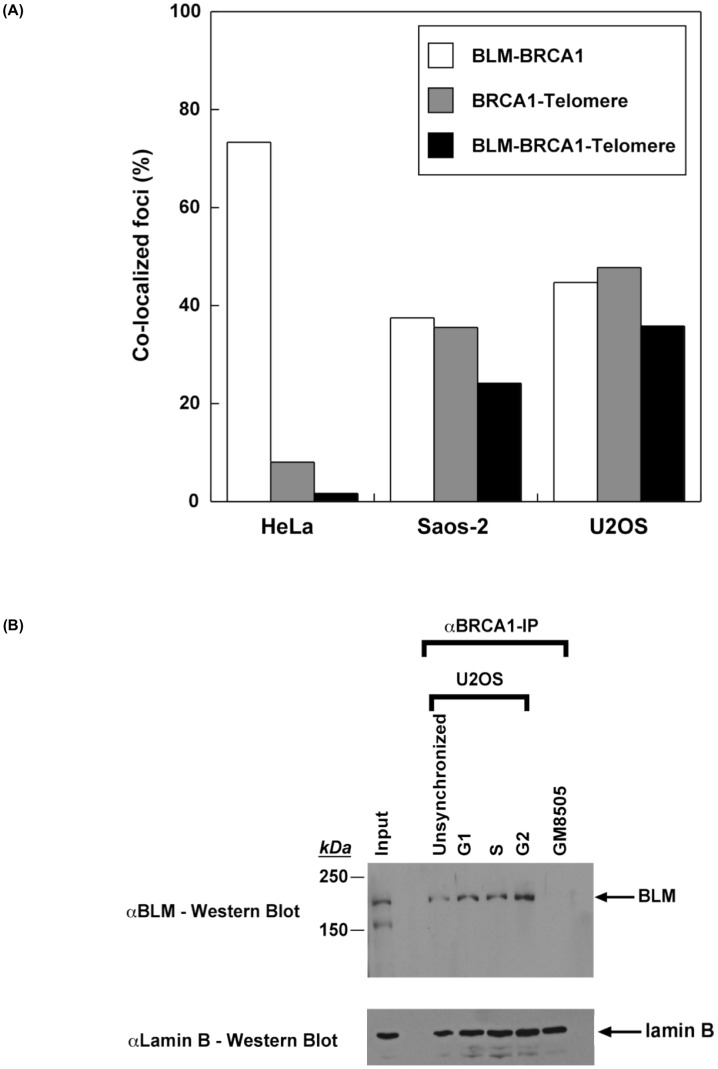
BRCA1 and BLM co-localize at telomeres only in ALT cells and physically interact predominantly during G2. (**A**) Quantitation of percent of cells with BLM-BRCA1, BRCA1-telomere, and BLM-BRCA1-telomere co-localized foci in Figure 3. (**B**) Upper panel: Co-immunoprecipitation of BLM with BRCA1 from U2OS cells. Cellular extracts (20 µg each) prepared at different stages of cell cycle were immunoprecipitated with an αBRCA1 antibody, separated on an 8% SDS-PAGE and analyzed by western blotting with αBLM antibody. Lower panel: Cellular extracts (20 µg each) used in upper panel corresponding to each stage were separated on an 8% SDS-PAGE and analyzed by western blotting with αLamin B antibody to show equivalent loading. The arrow mark indicates the position of BLM or Lamin B on the blot. Extracts from BLM-deficient cell line GM8505 served as a control.

BLM is critical for telomere maintenance and its loss results in telomere dysfunction, often yielding increase in telomeric associations [38]. BLM is found with BRCA1 in a variety of protein complexes associated with DNA damage. In order to assess its role in the context of telomeres, cells were synchronized as above and assessed for co-localization of BLM and BRCA1 at telomeres using the telomeric Cy3- (CCCTAA)_3_ PNA probe (Figure 3, 4A). Our results show that BLM and BRCA1 co-localize at telomeres only in ALT cells and that this interaction increases during G2. Furthermore, almost all BRCA1 foci at ALT telomeres include BLM (Figure 3, 4A). Telomerase-positive cells (HeLa) also contained BLM-BRCA1 co-localized foci (Fig 3, 4A), but none are found at telomeres. Similar results were obtained with another telomerase-positive cell line, MG63 (data not shown). These results demonstrate specific associations of BLM and BRCA1 at telomeres in ALT cells.

Since APB formation is thought to be integral to telomere maintenance in ALT cells, we assessed whether BLM and BRCA1 co-localize with PML at telomeres. While BLM commonly co-localizes with PML at telomeres, BRCA1 does not (Figure S2). As BRCA1 localization is dependent upon initiation of recombination events including DNA strand breaks, the absence of significant co-localization with PML at telomeres may indicate a transient association or exclusion of BRCA1 association from APBs during telomere metabolism. Association of BLM and BRCA1 was confirmed by co-immunoprecipitation of these proteins from total cell extracts made from ALT-positive U2OS cells at different stages of the cell cycle (Figure 4B). Our results show that BRCA1 co-immunoprecipitation with BLM is more pronounced at G2 compared to earlier stages. Extracts prepared from BLM-deficient cells served as a negative control. These data corroborate our immunofluorescence studies.

**Figure 5:**
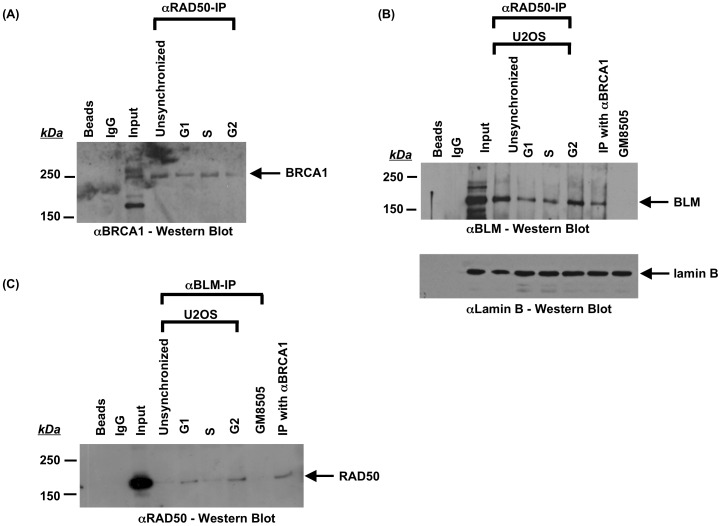
Co-immunoprecipitations of BRCA1, RAD50 and BLM from U2OS cells at different phases of the cell cycle. Cells were synchronized by double-thymidine block and extracts made at different cell cycle phases. Extracts were immunoprecipitated using indicated antibodies, separated by 8% SDS-PAGE and analyzed by western blotting using indicated antibodies. **(A and B) Immunoprecipitation of BRCA1 and BLM by RAD50.** Immunoprecipitation was performed using αRAD50 antibody on extracts representing unsynchronized cells or cells at G1-, S- and G2-phases of cell cycle. Controls included immunoprecipitation in the absence of αRAD50 antibody (Beads) or immunoprecipitation using IgG. Extracts in the absence of immunoprecipitation were included in the gels as input. Western blotting was performed using αBRCA1 (panel A) or αBLM antibody (panel B). Cellular extracts used for immunoprecipitation corresponding to each stage were separated on an 8% SDS-PAGE and analyzed by western blotting with αLamin B antibody to show equivalent loading (panel B lower blot). **(C) Immunoprecipitation of RAD50 by BLM.** Immunoprecipitation was performed using αBLM antibody on extracts representing unsynchronized cells or cells at G1-, S- and G2-phases of cell cycle. Controls included immunoprecipitation in the absence of αRAD50 antibody (Beads), immunoprecipitation using IgG or immunoprecipitation of extracts from a BLM-deficient cell line (GM8505). Extracts in the absence of immunoprecipitation were included in the gel as input. Western Blotting was performed using αRad50 antibody.

**Figure 6:**
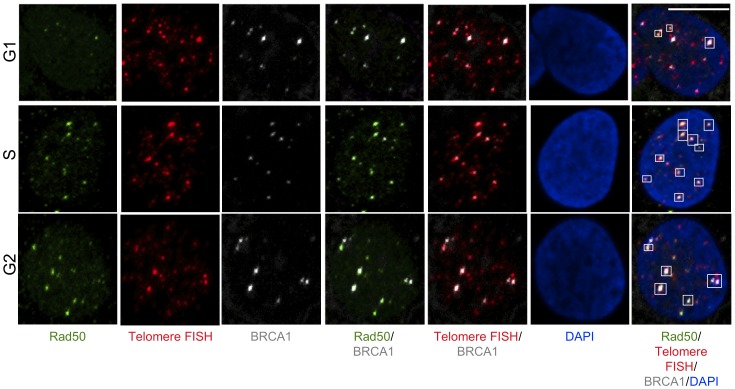
Co-localization of BRCA1 and RAD50 at telomeres at different stages of cell cycle in ALT cells. U2OS cells were synchronized by double thymidine block in G1-, S- and G2-stages of the cell cycle. Cells were fixed and stained with antibodies to BRCA1 (grey), Rad50 (green) and telomeres labeled by FISH with a PNA probe (Red). Nuclei are stained with DAPI. Images are shown as MIP image. White boxes indicate co-localizations of BRCA1, Rad50 and telomere FISH. Scale bar: 10 µm.

**Figure 7:**
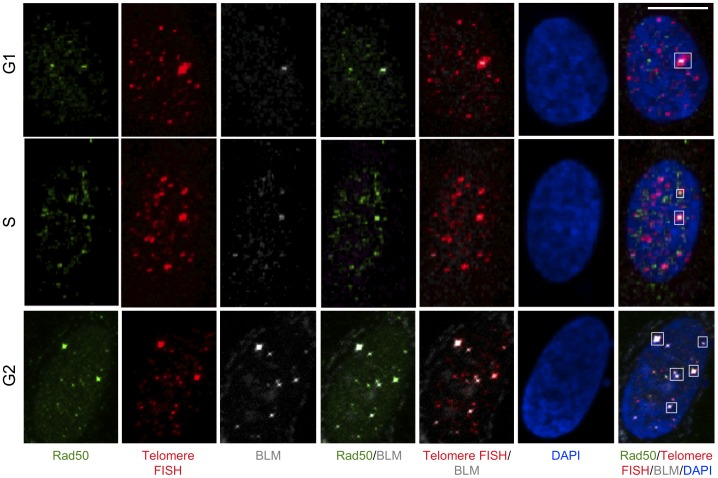
Co-localization of BLM and RAD50 at telomeres at different stages of cell cycle in ALT cells. U2OS cells were synchronized by double thymidine block in G1-, S- and G2-stages of the cell cycle. Cells were fixed and stained with antibodies to BLM (grey), Rad50 (green) and telomeres labeled by FISH with a PNA probe (red). Nuclei are stained with DAPI. Images are shown as MIP image. White boxes indicate co-localizations of BLM, Rad50 and telomere FISH. Scale bar: 10 µm.

**Figure 8:**
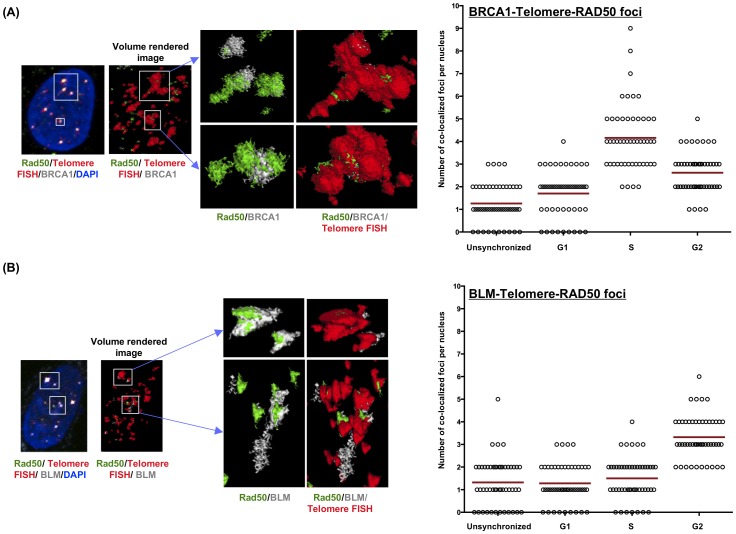
BRCA1 and BLM co-localize at telomeres along with RAD50 at different phases of cell cycle in ALT cells. U2OS cells were synchronized and stained with antibodies to BRCA1 or BLM (grey), Rad50 (green) and telomeres were labeled by FISH with a PNA probe (Red); nuclei were stained with DAPI. Representative images are shown as MIP image. White boxes indicate co-localizations of BRCA1 (**panel A**) or BLM (**panel B**) with Rad50 and telomere FISH. Co-localizations were confirmed by 3D rendering of multiple channels (volume rendering) using Metamorph analysis software. Enlargements of rendered co-localizations are shown at the right side and indicated by arrow. Co-localized foci of BRCA1 (or BLM), Rad50 and telomere were quantified per nucleus in unsynchronized, G1, S and G2 synchronized U2OS cells and represented graphically (right side of each panel). Each open circle represents an individual cell. Red bar indicate mean values.

The MRN (MRE11-RAD50-NBS) complex plays a central role in recruitment of BRCA1 to DNA double strand breaks (DSBs) [42], [48], [53], [61], [62], followed by the recruitment of other recombination proteins to initiate end-resection and strand exchange during recombination. MRN also promotes recruitment of BLM to DSBs to participate in early recombination events [63]–[65]. Our data show that BLM and BRCA1 physically interact and the BLM-BRCA1-telomere co-localized foci are specific to ALT cells (Figures 1-4). In order to discern whether BLM is present during early recombination events in ALT cells, U2OS cell extracts were prepared at different stages of the cell cycle and proteins immunoprecipitated using anti-RAD50. As expected, BRCA1 and RAD50 co-immunoprecipitate from these extracts predominantly in S-phase (Figure 5A). BLM also co-immunoprecipitates with RAD50 with an interaction that is more pronounced in G2-phase (Figure 5B,C). In order to confirm these interactions in the context of telomeres, U2OS cells were synchronized as before and assessed for co-localization of BRCA1 or BLM with RAD50 at telomeres using immunofluorescence and telomere FISH with a telomeric probe (Figures 6–8). Quantitation of co-localized foci confirmed association of BRCA1 with RAD50 at telomeres predominantly at S-phase (Figure 6, Figure 8A) and that of BLM with RAD50 at telomeres predominantly at G2-phase (Figure 7, Figure 8B). These results suggest that BLM and BRCA1 may be part of complexes distinct from those with BRCA1-MRN and may function at different stages of telomeric recombination [62]. In summary, cytological data suggest that BLM and BRCA1 co-localize to telomeres with other recombination proteins in ALT cells and at times during the cell cycle when ALT is thought to occur.

### BRCA1 stimulates BLM unwinding activity on a telomeric fork

**Figure 9:**
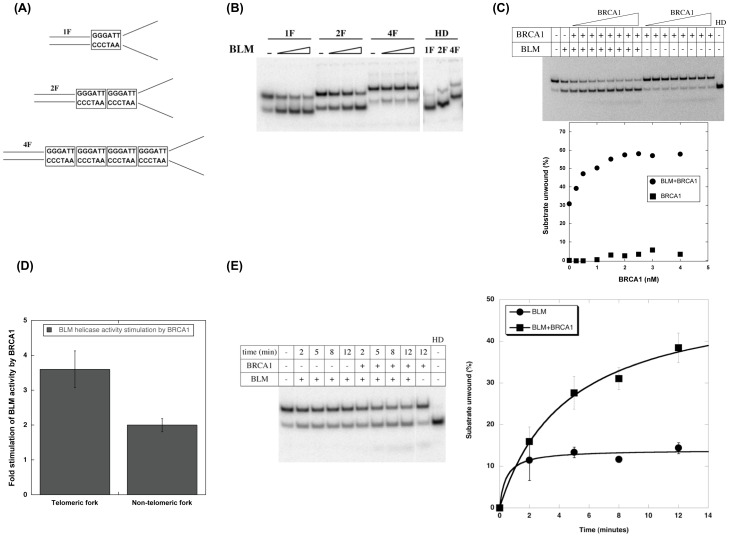
BRCA1 stimulates BLM helicase activity on telomeric repeat forks. (**A**) Fork substrates (1F, 2F and 4F) used for the study. The number of telomeric repeat units (GGGATT) are boxed in each fork substrate – 1F contains one repeat, 2F contains two repeats and 4F contains four repeats. (**B**) BLM helicase activity on fork substrates. Increasing amounts of BLM (0.6 nM, 1.2 nM and 4.2 nM) were used for each substrate, reactions were terminated after 12 minutes and analyzed by native PAGE. HD refers to heat-denatured substrate. (**C**) Effect of BRCA1 on BLM helicase activity on 2F substrate. Helicase reactions were performed for 12 minutes with 1.2 nM BLM and increasing concentrations of BRCA1 (0–4 nM). Reactions were terminated and analyzed by native PAGE. The amount of substrate unwound (%) was plotted as a function of BRCA1 (nM). A representative gel and corresponding graph are shown. (**D**) Stimulation of BLM activity by BRCA1 on telomeric and non-telomeric substrates. BLM helicase activity was tested in the presence of 3 nM BRCA1 on fork substrates that contained two telomeric repeats (panel A) or a non-telomeric sequence instead of the repeat units. Reactions were analyzed by native PAGE and quantitated on a phosphorimager. The fold stimulation of BLM activity by BRCA1 for each substrate is shown as histogram. (**E**) BRCA1 enhances the rate of unwinding of telomeric fork by BLM. Kinetics of BLM unwinding was analyzed in the presence of 1.2 nM BLM and 3 nM BRCA1. Reactions were analyzed as in C. The amount of substrate unwound (%) was plotted as a function time (minutes) and fitted to Michaelis-Menton kinetics using Kaleidagraph. A representative gel and corresponding graph are shown.

We asked whether there is any functional significance of BLM-BRCA1 using helicase assays with purified full-length BRCA1 and BLM proteins and telomeric substrates containing one, two or four telomeric repeats (TTAGGG). As unwinding by BLM was most prominent on the telomeric fork substrate (data not shown), it was used for further experiments (Figure 9A). Different concentrations of BLM were tested for unwinding of telomeric fork substrates in 12 minutes (Figure 9B). The fork substrate containing one telomeric repeat was unwound very effectively (>80%) at the lowest concentration of protein, whereas the fork substrate containing four telomeric repeats was unwound the least (Figure 9B). Subsequently, the fork substrate containing two telomeric repeats was used to measure the effect of BRCA1 on BLM unwinding (Figure 9C). The concentration of BLM was adjusted (1.2 nM) to achieve ≤30% unwinding in 12 minutes. The effect of BRCA1 on BLM helicase activity was then measured by addition of increasing concentrations of purified full-length BRCA1 (0–4 nM). DNA products were resolved by 10% native polyacrylamide gel electrophoresis (PAGE), and the percent of substrate unwound calculated and represented graphically as a function of BRCA1 concentration (Figure 9C). BLM activity is significantly enhanced approximately three-fold by BRCA1 in a protein concentration-dependent manner (Figure 9C). BRCA1 alone did not have a significant unwinding activity even at high concentrations (Figure 9C gel inset). Our data indicate that BRCA1 increases BLM unwinding activity on telomeric fork substrates. The effect of BRCA1 on BLM helicase activity was also tested using a non-telomeric fork. Our data indicate that full-length BRCA1 stimulates BLM on a non-telomeric fork albeit less than that on a telomeric fork (Figure 9D; ∼3.6-fold increase for telomeric substrate vs ∼1.9-fold increase for non-telomeric substrate). Stimulation of BLM activity by BRCA1 on a non-telomeric fork agrees with previously published work using an internal truncated fragment (amino acids 452–1079) of BRCA1 [58]. These data also suggest that the stimulation of BLM using non-telomeric substrates is independent of the RING and BRCT domains of BRCA1 and by extension, of BARD1 or other protein partners that utilize the BRCT domain to interact with BRCA1 [46], [58]. These results indicate that full length BRCA1 is sufficient to stimulate BLM unwinding of telomeric forked DNA although it is unknown whether this would be enhanced by BARD1 or other BRCA1 partners.

### BRCA1 increases the BLM unwinding rate on a telomeric fork

Finally, to understand the mechanism of action of BRCA1 on BLM, we assessed unwinding kinetics. Reactions were assembled on ice in the presence of BRCA1, started by BLM addition, stopped at regular time intervals and unwinding analyzed by native PAGE. BRCA1 stimulates BLM in a time-dependent manner (Figure 9E). The respective rates of reaction were calculated by plotting the substrate unwound in the initial stages of reaction against time. The BLM initial unwinding rate was increased by 1.7-fold in the presence of BRCA1.

These biochemical experiments indicate that BRCA1 effectively increases the rate of unwinding by BLM to resolve fork substrates that contain small stretches of telomeric repeats. The lack of resolution of fork substrates containing four repeats may reflect the lack of processivity of BLM rather than an effect of BRCA1 or a possible requirement of additional factors to overcome the energetic barrier imposed by a stretch of G-rich duplex regions. Our data do not address the temporal requirements of the BLM-BRCA1 interaction. However, their enhanced association at G2 suggests a function during late replication or during recombination-associated events at the telomere [38], [66]. While BRCA1 is essential for recruitment of recombination proteins to promote strand processing and invasion, and to form recombination intermediates, its recruitment to telomeres requires RAD50 during S-phase [42], [53], [61]. Our data indicate that BLM is also part of stable complexes with RAD50, albeit predominantly in G2 and suggest that BLM-BRCA1 complexes may be distinct from BRCA1-RAD50 complexes required during recombination initiation (Figures 5–8). It is also unclear whether resolution of the intermediates by BLM requires the recruitment of BRCA1 or the realignment of existing BRCA1-containing complexes. The apparent opposing effects of BRCA1 in promoting recombination to resolve DNA damage through formation of recombination intermediates and preventing recombination by facilitating repair through resolution of recombination intermediates with BLM is intriguing. Given the importance of recombination in ALT, an inhibitory activity of BRCA1 might be essential to limit recombination at telomeric regions resulting from a strand break due to damage or replicative stress. Such a function is consistent with an increase in telomeric chromosomal end-to-end fusions and telomere dysfunction that is seen with deficiency of BRCA1 [25], [28], [38]-[40], [51], [52], [54]. Our data implicate both BRCA1 and BLM in alternative telomere length maintenance mechanisms and provide an explanation for how recombination of telomeric repeats could be regulated by BLM and BRCA1 interactions at telomeres.

## Materials and Methods

### Cell lines

MG63 (osteosarcoma, ATCC), HeLa (cervical adenocarcinoma, ATCC), U2OS (osteosarcoma, ATCC) and Saos-2 (osteosarcoma, ATCC) cells were cultured in Dulbecco's modified Eagle's minimal essential medium (DMEM) (Invitrogen) supplemented with 10% fetal bovine serum (FBS) (Hyclone). All cultures were grown without antibiotics in a humidified incubator at 37°C with 5% CO_2_.

### Antibodies and *siRNA*

Antibodies for immunofluorescence included: BRCA1 (A301-378A, Bethyl Laboratories; OP92, Calbiochem), and PML (PG-M3, Santa Cruz; ab53773, Abcam). Antibodies for western blot included: BRCA1, RAD50 (Santa Cruz), TRF2 (Imgenex) and lamin B (C-20, Santa Cruz). The BLM antibody was a generous gift from Dr. Albert Davalos. *BRCA1 siRNA* (ON target plus Human *BRCA1* (672) *siRNA* SMARTpool) was purchased from Thermo Scientific.

### Cell synchronization

Cells were plated at 30% confluence and treated with 2 mM thymidine (Sigma) for 18 h (first block). After the first thymidine block, cells were washed with PBS and released for 9 h by adding DMEM containing 10% FBS. After release, cells were again treated with 2 mM thymidine for 17 h (second block). After the second block, cells were washed with PBS and released by adding DMEM containing 10% FBS. Cells for G1 analysis were collected at this point; cells for S-phase analysis were typically collected after 3 h post release and G2 analysis were collected after 7 h post release.

### Cell cycle analysis

Cells were collected and fixed with ice-cold 80% (v/v) ethanol for 30 min on ice. Cell were washed twice with PBS containing 0.1% EDTA (w/v) and collected by centrifugation. Cells were re-suspended in 0.03 mg/ml propidium iodide (Sigma) and 0.3 mg/ml RNAse A (Sigma) and incubated at 4°C for 1 h. DNA content was analyzed using a FACSCalibur flow cytometer (BD Biosciences).

### Immunofluorescence and telomere FISH

Unsynchronized, synchronized and *siRNA*-treated cells were grown on coverslips and fixed for 10 min in PBS with 4% (v/v) paraformaldehyde. Cells were dehydrated in a graded ethanol series (70% (v/v) for 3 min, 90% (v/v) for 2 min and 100% for 2 min) and rehydrated for 30 minutes in consecutive washes with PBS and 2X SSC. Cells were overlaid with 0.6 µg/ml Cy3-(CCCTAA)_3_ PNA probe (Panagene) in *in situ* hybridization solution (Enzo Life Sciences), denatured at 80°C for 3 min and hybridized at 37°C for 3 h. Cells were washed with 2X SSC and PBST. Cells were then permeabilized for 10 min in PBS with 0.5% Triton X-100 (v/v) and blocked for 30 min in 10% goat serum/PBST (0.1% Tween-20 in PBS) (v/v) at room temperature. Cells were incubated with primary antibody diluted in 1% BSA/PBST for 1 h at room temperature or at 4°C overnight, washed with PBST, incubated with secondary antibody diluted in 1% BSA/PBST for 1 h at room temperature and washed with PBST. Finally, cells were rinsed in PBS and mounted onto glass slides in mounting media with DAPI (Vector Laboratories, Inc.). Slides were analyzed with an Olympus FV 1000 spectral confocal system at the Ohio State Campus Microscopy and Imaging Facility or with a Zeiss Axioskop. Four color imaging was performed using Olympus FV1000 Filter confocal system. Nuclei were captured in 15–20 Z plane increments of 0.44 µm and merged using the extended focus maximum intensity projection (MIP) settings. The 3D co-localization rendering of multiple channels (volume rendering) was created using Metamorph analysis software. At least 50 cells per group were analyzed to generate the percentage of cells with co-localized foci. Similar results were observed in several independent experiments.

### Western blot and immunoprecipitation

Western blot analysis was performed using standard methods [67], [68]. Immunoprecipitation (IP) was performed at 4°C. For the co-immunoprecipitation assay, protein lysates were prepared from unsynchronized and synchronized U2OS cells in NP40 lysis buffer (50 mM Tris.HCl pH 8.0, 150 mM NaCl, 2 mM EDTA, 1% NP40 + protease inhibitors). Equal amount of protein lysate (20 µg) was pre-cleared using protein-A sepharose beads (GE Healthcare Biosciences) for 30 minutes, then applied to protein-A beads that were incubated with either 10 µl of anti-BRCA1, anti-BLM, anti-Rad50 antibody, rabbit-IgG or mouse-IgG. Protein-A-bound immune complexes were washed three times with IP wash buffer (20 mM Tris.HCl pH 8.0, 2 mM EDTA, 50 mM NaCl); suspended in SDS-sample buffer followed by boiling for 5 minutes. Immunoprecipitates were separated on 6% SDS-PAGE gel and transferred to a PVDF membrane. The membrane was blotted overnight at 4°C with either anti-BRCA1, anti-BLM or anti-Rad50.

### Analyses

Dot plots were generated using Graph Pad Prism.

### Protein purification

His-tagged human BLM was expressed in yeast and purified as described previously [67], [68]. Recombinant full length BRCA1 was purchased from Active Motif.

### Oligonucleotides

Oligonucleotides containing different telomeric repeat units and the corresponding complementary strands were designed as described and purchased from Operon [69]. The non-telomeric substrate was made by annealing the following strands: 5′-TTTTTTTTTTTTTTT**TTCAGTAGATCA**CATGCACTAC-3′ and 5′-GTAGTGCATG**TGATCTACTGAA**TTTTTTTTTTTTTTT-3′.

### Helicase assays

The repeat-containing oligonucleotide of each substrate was labeled, annealed with its complementary oligonucleotide and purified on 10% native PAGE. Helicase assays were performed as described [67], [68] in the following buffer: 20 mM Tris-HCl pH7.5, 5 mM MgCl_2_, 1 mM DTT, 2 mM ATP, 100 mM NaCl and 100 µg/ml BSA. Reactions (20 µl) were assembled on ice in the presence (or absence) of BRCA1 and 5fmol of respective labeled substrate. Reactions were started with the addition of BLM and incubated at 37°C for 12 minutes (or as indicated for kinetics). Reactions were stopped by addition of 20 µl of 2X stop buffer containing 1 pmol/ul unlabeled oligonucleotide (corresponding to the labeled oligonucleotide). Reactions were analyzed by a 10% native PAGE in 1X TBE. Gels were dried, exposed on a phosphorimager plate and analyzed by ImageQuant software on a Typhoon phosphorimager. Bands were quantitated, amount of substrate unwound calculated and plotted as a function of protein concentration or time as indicated. Plots were fitted to Michaelis-Menton kinetics as indicated using Kaleidagraph software.

## Supporting Information

Figure S1 *BRCA1 siRNA* reduces BRCA1 staining and localization at telomeres. (**A**) Western blot analysis of BRCA1 and lamin B (loading control) in 25 µg of protein extracted from MG63 and U2OS cells treated with mock, scrambled or BRCA1 siRNAs. (**B**) MG63 and U2OS cells were stained with antibodies to BRCA1 (green), telomeres labeled by FISH with a PNA probe (red), nuclei were labeled with DAPI (blue) after 72 hours of *BRCA1 siRNA* knockdown. (**C**) Quantitation of BRCA1 and telomere co-localizations per nucleus in scrambled and BRCA1 siRNA-treated MG63, HeLa, U2OS and Saos-2 cells. Each triangle (TA+ cells) or open circle (ALT cells) represents an individual cell. Red bars indicate mean values.
(TIFF)

Figure S2 PML co-localizes with BLM but not BRCA1 at ALT telomeres. Cells were synchronized and stained with antibodies to BRCA1 (green), BLM (white) or PML (top: white; bottom: green), telomeres were labeled by FISH with a PNA probe (red), and nuclei were stained with DAPI (blue). Yellow arrows indicate foci with all three signals.
(TIFF)
