# Supplementary figures and images for: Association of BLM and BRCA1 during Telomere Maintenance in ALT Cells
Source: PLoS One. 2014 Aug 1;9(8):e103819. doi: 10.1371/journal.pone.0103819 (PMC4118958; doi:10.1371/journal.pone.0103819)

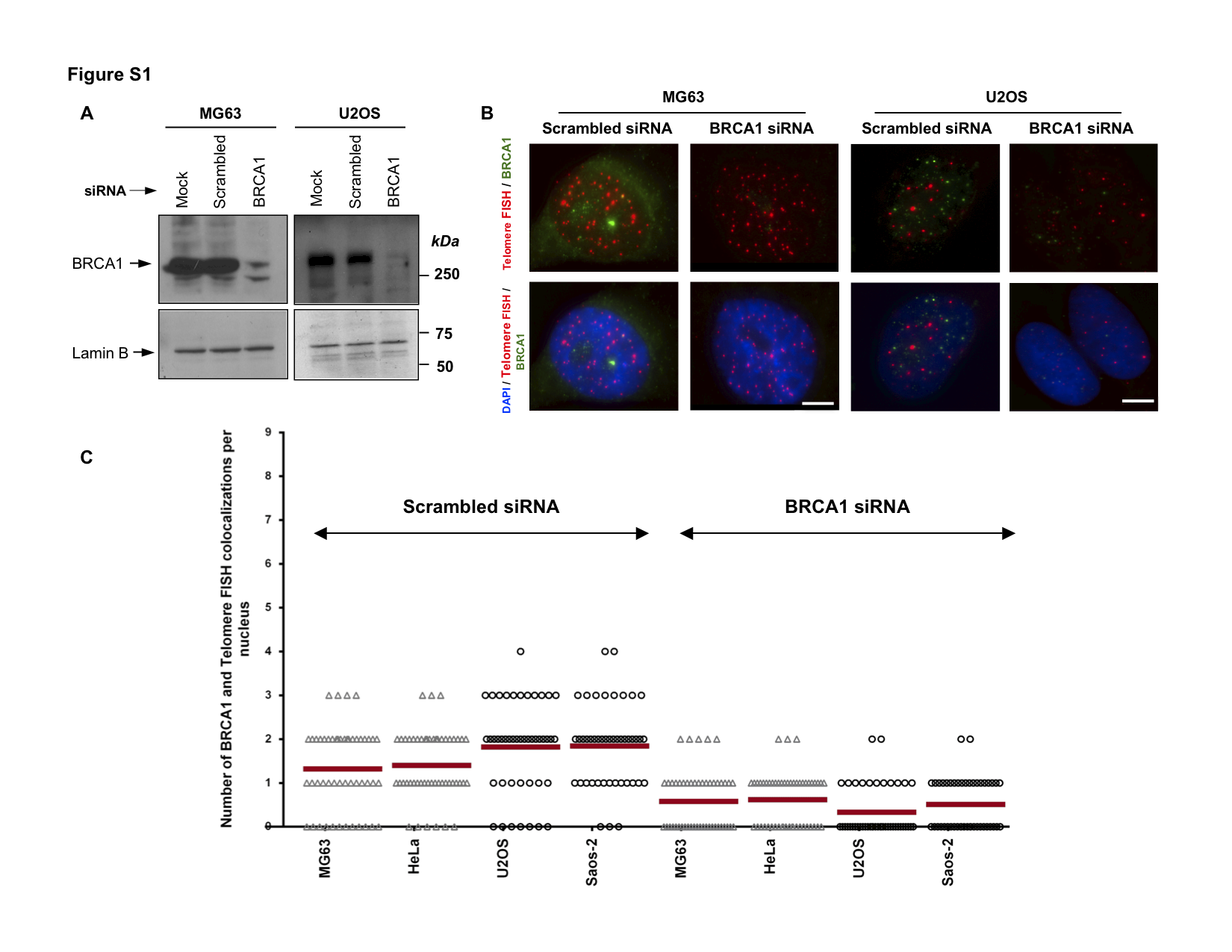

Supplement: Figure S1 — BRCA1 siRNA reduces BRCA1 staining and localization at telomeres. (A) Western blot analysis of BRCA1 and lamin B (loading control) in 25 µg of protein extracted from MG63 and U2OS cells treated with mock, scrambled or BRCA1 siRNAs. (B) MG63 and U2OS cells were stained with antibodies to BRCA1 (green), telomeres labeled by FISH with a PNA probe (red), nuclei were labeled with DAPI (blue) after 72 hours of BRCA1 siRNA knockdown. (C) Quantitation of BRCA1 and telomere co-localizations per nucleus in scrambled and BRCA1 siRNA-treated MG63, HeLa, U2OS and Saos-2 cells. Each triangle (TA+ cells) or open circle (ALT cells) represents an individual cell. Red bars indicate mean values. (TIFF) [file pone.0103819.s001.tif]

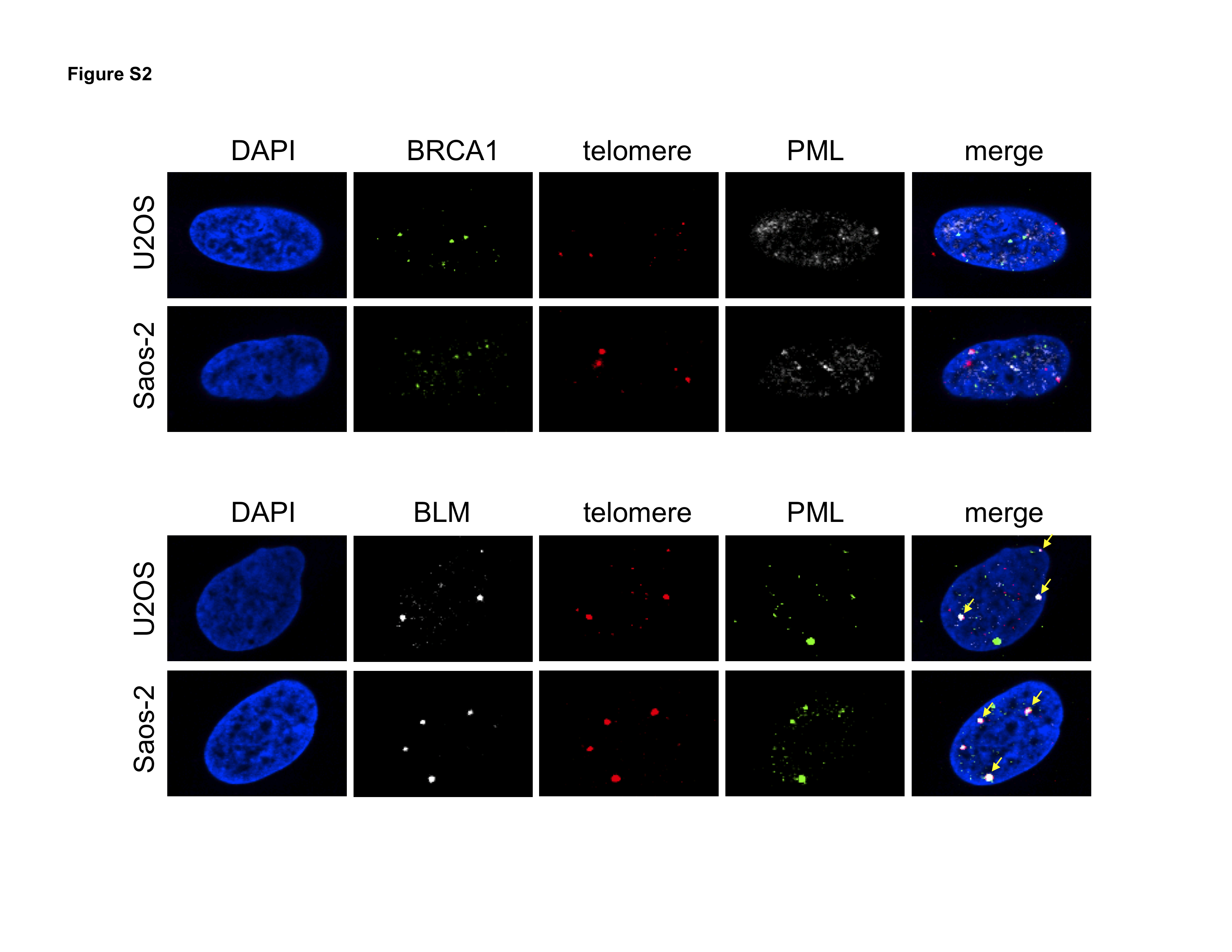

Supplement: Figure S2 — PML co-localizes with BLM but not BRCA1 at ALT telomeres. Cells were synchronized and stained with antibodies to BRCA1 (green), BLM (white) or PML (top: white; bottom: green), telomeres were labeled by FISH with a PNA probe (red), and nuclei were stained with DAPI (blue). Yellow arrows indicate foci with all three signals. (TIFF) [file pone.0103819.s002.tif]
